# The “CPC Clip Motif”: A Conserved Structural Signature for Heparin-Binding Proteins

**DOI:** 10.1371/journal.pone.0042692

**Published:** 2012-08-06

**Authors:** Marc Torrent, M. Victòria Nogués, David Andreu, Ester Boix

**Affiliations:** 1 Department of Biochemistry and Molecular Biology, Biosciences Faculty, Universitat Autònoma de Barcelona, Cerdanyola del Vallès, Spain; 2 Department of Experimental and Health Sciences, Universitat Pompeu Fabra, Barcelona Biomedical Research Park, Barcelona, Spain; Russian Academy of Sciences, Institute for Biological Instrumentation, Russian Federation

## Abstract

Glycosaminoglycans (GAGs) are essential molecules that regulate diverse biological processes including cell adhesion, differentiation, signaling and growth, by interaction with a wide variety of proteins. However, despite the efforts committed to understand the molecular nature of the interactions in protein-GAG complexes, the answer to this question remains elusive.

In the present study the interphases of 20 heparin–binding proteins have been analyzed searching for a conserved structural pattern. We have found that a structural motif encompassing one polar and two cationic residues (which has been named the *CPC clip motif*) is conserved among all the proteins deposited in the PDB. The distances between the α carbons and the side chain center of gravity of the residues composing this motif are also conserved. Furthermore, this pattern can be found in other proteins suggested to bind heparin for which no structural information is available. Hence we propose that the CPC clip motif, working like a staple, is a primary contributor to the attachment of heparin and other sulfated GAGs to heparin-binding proteins.

## Introduction

Glycosaminoglycans (GAGs) are negatively charged polysaccharides, with molecular weights ranging from 10 to 100 kDa, composed of repeating units of uronic acid (D-glucuronic or L-iduronic acid) and amino sugars (D-galactosamine or D-glucosamine). D-glucosamine-containing GAGs, like heparin, are named glucosaminoglycans [1].

GAGs have central biological functions including wound healing [2], anti-coagulation [3], cell signaling, development and angiogenesis [4], tumor progression and metastasis [5] and can even play an important role in amyloid-related diseases [6]. In particular, heparin can preclude blood clotting and is mainly used as anti-coagulant for the treatment of thrombosis, thrombophlebitis and embolism [3]. Additionally, GAGs are involved in cell proliferation and diseases such as rheumatoid arthritis, inflammatory bowel disease and infections associated with inflammatory responses [7] and can hinder HIV-1 or herpes simplex virus activity through binding to the viral surface glycoproteins [8].

In this context, heparin and heparan sulfate (HS) have been found to bind a wide variety of proteins with diverse functions, including growth factors, thrombin, chemokines and viral proteins [1], [9]. Unfortunately, despite the growing pharmaceutical interest in protein-sugar interactions, structural requirements for GAG binding are still not well characterized [1], [10], [11].

Cardin and Weintraub analyzed the structure of heparin-binding proteins and proposed that typical binding sites contain the sequence-based motif XBBXBX or XBBBXXBX, where B is a lysine or arginine (rarely His) and X a hydropathic residue such as Ala, Gly, Ile, Leu or Tyr [12]. In this way, a conserved sequence (XBBBXXBBBXXBBXBX) was similarly proposed for the Von Willebrand factor and a TXXBXXTBXXXTBB sequence was ascribed to α and β fibroblast growth factors (αFGF, βFGF) and transforming growth factor β-1 (TGFβ-1) [13].

Heparin-binding domains always contain cationic residues, which bind to anionic (carboxylate, sulfate) groups in heparin through electrostatic and hydrogen-bonding interactions. It has been reported that a precise spacing of cationic clusters is required, and efficient heparin-binding peptides were designed on this basis [14]. However, the specificity of these interactions remains elusive and is still poorly understood [11].

Here we report a novel structural signature for heparin-binding proteins, which is conserved in all such protein structures available in the Protein Data Bank (PDB). The motif involves two cationic residues (Arg or Lys) and a polar residue (preferentially Asn, Gln, Thr, Tyr or Ser, more rarely Arg or Lys), with fairly conserved distances between the α carbons and the side chain center of gravity, defining a clip-like structure where heparin would be lodged. This structural motif is highly conserved and can be found in many proteins with reported heparin binding capacity.

## Methods

### Protein structure analysis

Protein three-dimensional structures were obtained from the Protein Data Bank (www.pdb.org). The summary of ligand interactions with IDS (2-O-sulfo-α-L-iduronic acid) and SGN (N,O6-disulfo-glucosamine) has been performed using the PDBeMOTIF web server (http://www.ebi.ac.uk/pdbe-site/pdbemotif/) and statistically evaluated using the Prism software. To define the CPC clip motif, the location of protein-ligand interfaces has been performed using the PISA web server (http://www.ebi.ac.uk/msd-srv/prot_int/pistart.html) and the residues involved in heparin binding were analyzed to outline a conserved motif. To further check the contacts detected by PISA, a manual inspection of the interacting residues was performed using Pymol (DeLano Scientific, San Carlos, CA), defining a 3.5 Å cut-off distance for hydrogen bond interactions and a 4.5 Å for electrostatic interactions. The analysis of α-carbon distances has also been calculated using Pymol. Chemokine domain structural modeling has been performed using the automated Swiss Model server (http://swissmodel.expasy.org/). Amino acid conservation was evaluated using the ConSurf web server (http://consurf.tau.ac.il/). Sequence alignments were drawn using ESPript (http://espript.ibcp.fr/ESPript/ESPript/) and figures were prepared using Pymol.

### Datasets

The discovery set contains 20 proteins crystallized with heparin analogs and were used to define the CPC clip motif (Table S1). All proteins included share less than 25% of sequence similarity with the remaining dataset, except for the pairs: 1FQ9/1E0O, 1AXM/1BFB and 1G5N/2HYU. The later were included because different binding sites were detected for those structures, probably reflecting complementary binding sites for larger heparin molecules. The testing dataset contains 48 proteins; 24 proteins with experimental evidence of heparin binding capacity not included in the discovery set (<50% sequence similarity; positive testing dataset) and 24 proteins with neither described not expected heparin binding activity (negative testing dataset; five negative testing datasets were designed). To select the structures belonging to the negative dataset, we have numbered each structure in the SPASM database [15] (23746 proteins) and selected 24 proteins among them using a random number generation function. Proteins with potential heparin binding capacity (e.g. growth factors, viral capsid proteins, etc. or proteins related to the discovery set) were excluded. Proteins in the negative dataset were ensured to have similar size distribution than in the positive dataset (p<0.05).

### CPC enrichment in heparin binding proteins

To test the CPC motif enrichment in heparin-binding proteins, the SPASM algorithm [15] has been used to search for CPC motif hits in the testing dataset (48 proteins) allowing 2.5 Å of RMSD. The process has been repeated five times with five independent negative testing datasets. In the SPASM computational algorithm, the residues conforming the motif are represented by its Cα atom and its side-chain center of gravity. SPASM calculates the distances between these pseudo-atoms, and identifies all sets of identical (or similar) residues for each protein database. Afterwards, the hits obtained were classified based on their database origin to determine the enrichment fraction.

### Molecular docking simulations

Docking simulations were conducted with AutoDock 4.2 (Scripps Research Institute. La Jolla, CA). The ligand used was the heparin dodesaccharide characterized by NMR and deposited in the PDB (code 1HPN). In all crystal structures used, water molecules were removed from the structure. Hydrogen atoms and atomic partial charges (using the Gasteiger method) were added using Autodock Tools. Protein was kept rigid. Heparin was allowed to bend by selecting ten degrees of freedom along the oligosaccharide backbone (Figure S1A). The interaction of a probe group, corresponding to each type of atom found in the ligand, within the whole protein structure, was computed at 0.500 Å grid positions in a box centered in the protein.

The docking was accomplished using 100 Lamarkian genetic algorithms (LGA) runs and the initial position of the ligand was random. The number of individuals in populations was set to 150. The maximum number of energy evaluations that the genetic algorithm should make was 2500000. Maximum number of generations was 27000. The number of top individuals that are guaranteed to survive into the next generation was 1. Rates of gene mutation and crossover were 0.02 and 0.80, respectively. Following docking, all structures generated for the same compound were subjected to cluster analysis, cluster families being based on a tolerance of 10 Å for an all-atom root mean square (RMS) deviation from a lower energy structure. A second docking stage was performed to increase performance and complete exploration of conformational space. Thus, the global minimum structure found in the previous run was subjected to redocking under the same conditions and to 2 Å cluster analysis, using a box centered in the ligand with a 0.375 Å of grid spacing. The global minimum structure found in this second-stage docking was considered as the final result.

To better estimate the binding energy for the complex, a heparin disaccharide (H3S, obtained from the PDB, code 1U4M) was docked using the parameters described before within a 50 Å^3^ grid box centered in the CPC motif detected. The heparin disaccharide was allowed to bend by allowing two degrees of freedom along its backbone (Figure S1B).

### Automated motif finding

The coordinates of the five identified canonic CPC clip motifs (C-Asn-C, C-Gln-C, C-Ser-C, C-Thr-C and C-Tyr-C, where C is a cationic residue, Arg or Lys) identified have been subjected to analysis using the SPASM algorithm (Lys and Arg were considered equivalent as cationic residues), then analyzed with SAVANT [16] and finally filtered using DEJANA [16]. SAVANT algorithm performs an all-atom least squares superpositioning of the query motif on each hit found by SPASM. To analyze the gene-related distribution of the proteins found with SPASM, PICR (http://www.ebi.ac.uk/Tools/picr/) has been used to convert PDB to SwissProt identifiers. The database generated was inspected using PANTHER (http://www.pantherdb.org/) and the genes identified were classified by gene function and GO annotation. CD-HIT was used to avoid check protein similarity (http://weizhong-lab.ucsd.edu/cdhit_suite/).

## Results and Discussion

### Structural analysis of heparin-binding proteins

**Figure 1:**
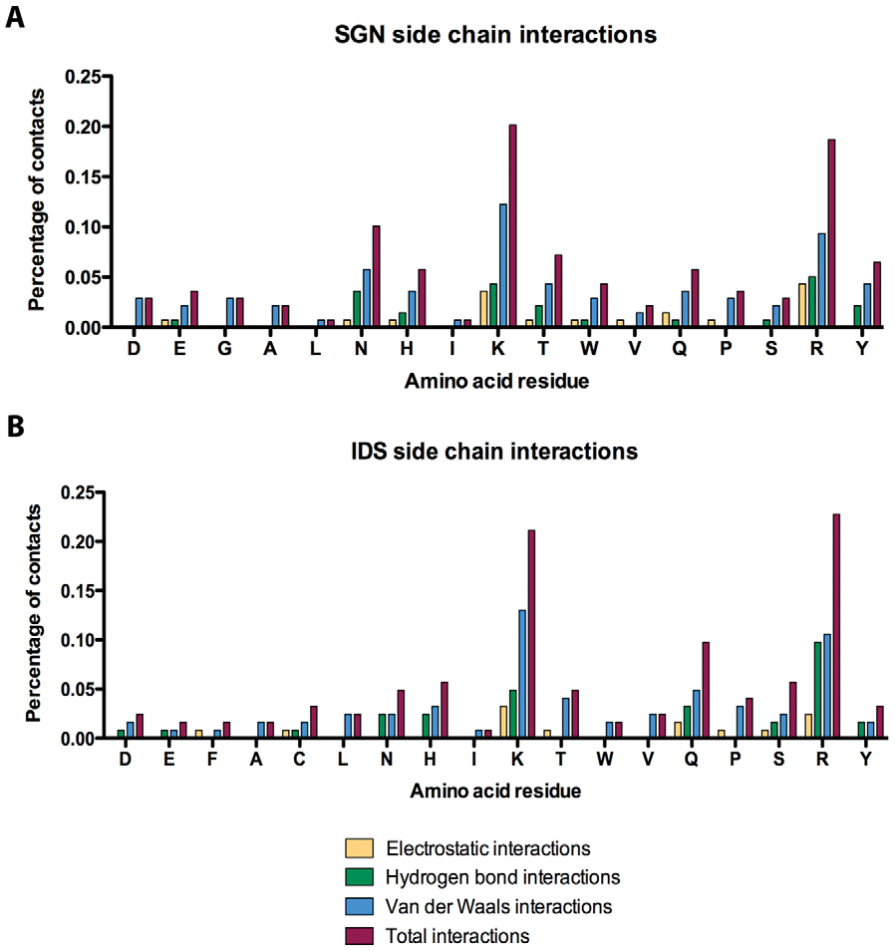
Summary of side-chain amino acid interactions for protein-heparin complexes deposited in the PDB. Molecular contacts were inspected for (A) SGN and (B) IDS, the two major components of heparin. The fraction of contacts is represented for each amino acid.

At present, 20 non-redundant three-dimensional protein structures in complex with heparin disaccharides or oligosaccharides have been deposited in the PDB. Using the PDBeMOTIF server we have examined them to identify the protein primary amino acid residues that interact with the two main components of heparin, N,O6-disulfo-glucosamine (SGN) and 2-O-sulfo-α-L-iduronic acid (IDS). We have inspected the amino acid side chains for hydrogen bonding, electrostatic and van der Waals interactions with heparin. The results (Figure 1) confirm that Arg and Lys are essential, making most of the electrostatic and hydrogen-bonding interactions with both IDS and SGN.

A high contribution for hydrogen bonding is also observed for Asn and Gln and, less frequently for Tyr, Ser and Thr. In fact, hydrogen-bonding contacts have been described to be the major contribution to heparin interaction in the bFGF [17] and the natriuretic peptide [18], together with electrostatic interactions. It can also be observed that Asn is preferred over Gln in SGN binding whereas the opposed trend is found for IDS, suggesting a favored role for the longer side chain of Gln in IDS interaction. Our analysis also shows that Van der Waals interactions are mainly confined to the side chains of either polar or cationic, with surprisingly little contribution from aromatic or hydrophobic residues.

According to Cardin-Weintraub, cationic Lys and Arg, on the one hand, and Ala, Gly, Ile, Leu or Tyr, on the other hand, would make the strongest contribution to heparin binding [12], [13]. Our analysis, however, shows that such composition underestimates the role of polar residues (mainly Gln and Asn) in heparin recognition (Figure 1).

### Definition of the CPC clip motif

**Figure 2:**
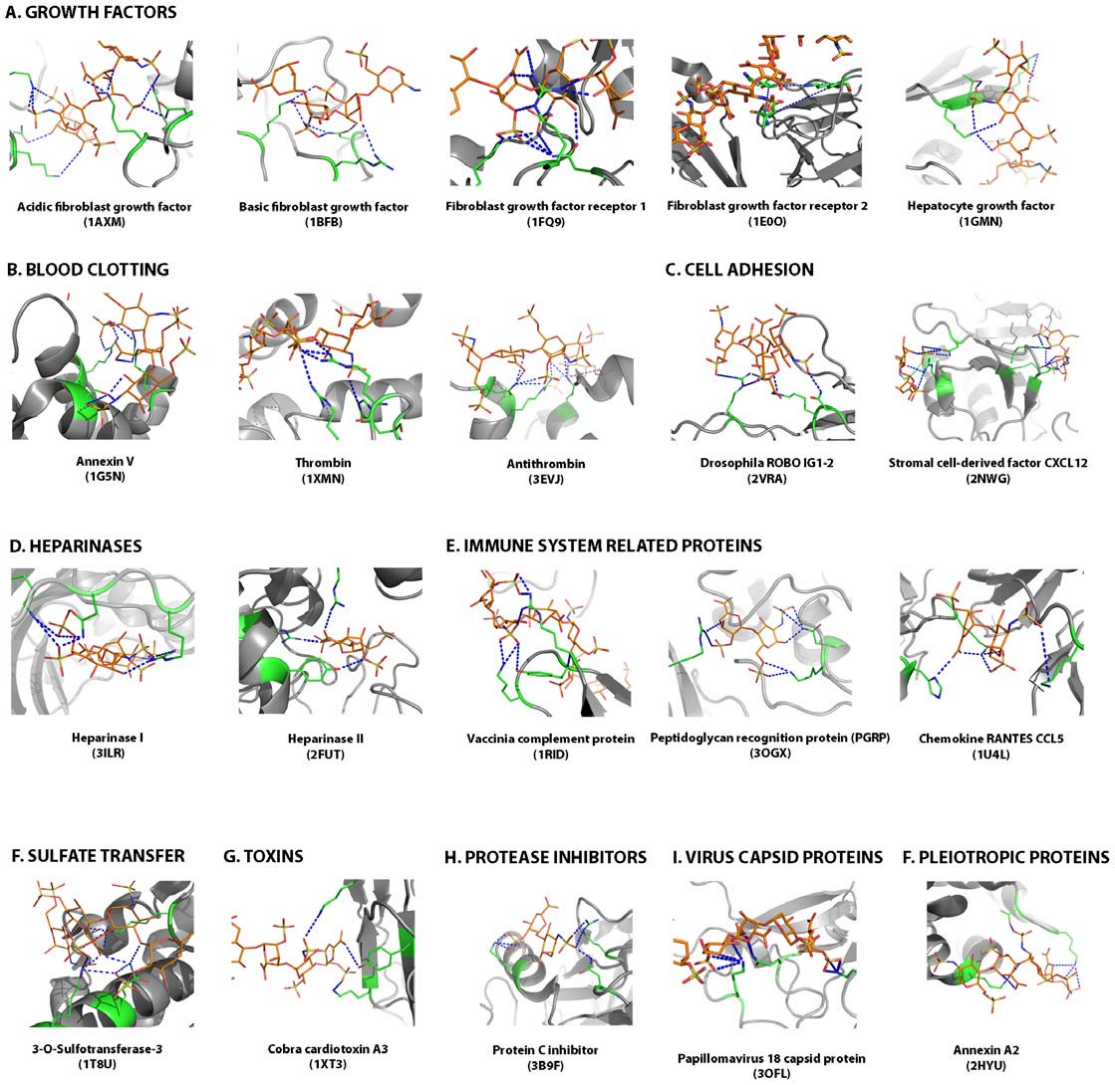
Molecular representation of the CPC clip motif for the 20 reference protein-heparin complexes. For each complex, the ligand is colored in orange, the amino acids belonging to the CPC clip motif in green and suggested polar interactions are depicted as blue dashed lines. Images were generated with Pymol.

**Figure 3:**
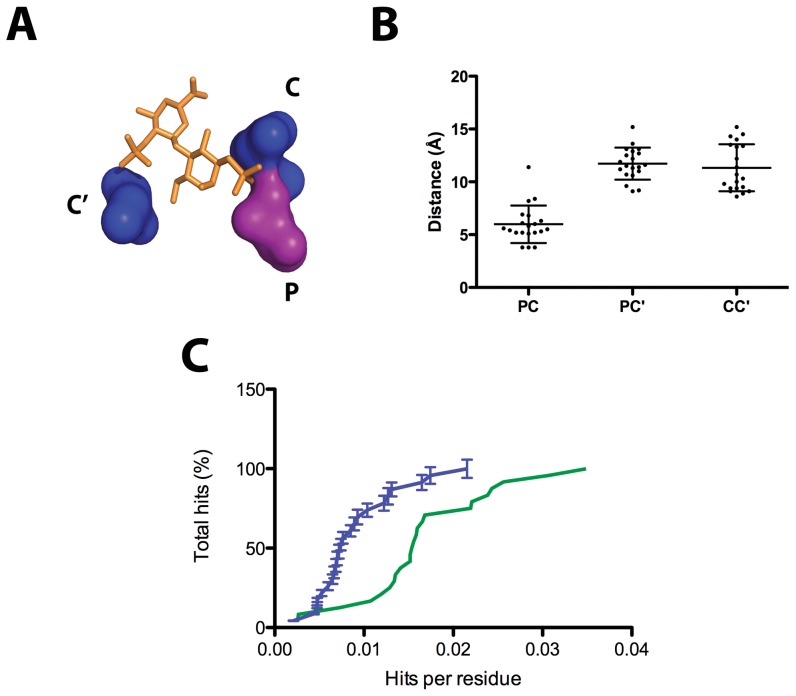
Statistical analysis of the Cα and side chain center of gravity distances between the amino acids conforming the CPC clip motif. (A) Schematic representation of the CPC clip motif, composed of one polar (P) and two cationic residues (namely C and C′, being C the closest to the polar residue). Image was generated with Pymol. (B) Measured PC, PC′ and CC′ distances for the 20 reference proteins described. (C) Enrichment of CPC clip motif in heparin-binding proteins. The negative and positive testing databases were analyzed by SPASM and a cumulative frequency histogram plotting the number of hits per residue is depicted. The positive testing dataset is colored in green and the negative dataset in blue. Each point in the negative dataset represents the average of five independent tests and errors bars are depicted. See the *Materials and Methods* section for further information.

We have used the PDBePISA server [19] to characterize hydrogen-bonding contacts in the ligand-protein interfaces of the discovery dataset. This analysis shows Arg and Lys residues making the primary hydrogen-bonding contacts. Polar residues, for their part, could fine-tune the precise recognition of GAGs. More specifically, detailed inspection of the interacting residues reveals a conserved pattern that comprises one polar and two positively charged residues (Figure 2) whose spatial arrangement allows defining regular distances between cationic (C and C′) and polar (P) residue α-carbons and side-chain center of gravity (Figure 3). Average measured distances are 6.0±1.8 Å (PC), 11.6±1.6 Å (PC′) and 11.4±2.4 Å (CC′) for Cα and 6.0±1.9 Å (PC), 10.6±1.8 Å (PC′) and 10.7±2.0 Å (CC′) for side-chains center of gravity. Hence our analysis suggests that a structural rather than a sequence pattern appears to be conserved in heparin-binding proteins. Additional contacts involving both side-chain and main-chain atoms can be found in the identified interfaces. These would provide complementary contacts for a fuller fastening of heparin. Thus, the cation-polar-cation (CPC) motif outlined above could be regarded as the minimum structural requirement for heparin binding in proteins. We should also consider that heparin-binding sites could be located in monomers but also in oligomeric interfaces. The CPC motif can thus be shared by two monomers. For example, in structure 1AXM a monomeric and a dimeric binding site can be described where one monomer contains one side of the motif (P and C residues) and the other the remaining residue (C′).

To test whether heparin-binding proteins are indeed enriched in the CPC clip motif, we have automatically searched for the motif in the testing dataset, which contains 48 proteins not related to the discovery set (24 proteins with experimental evidence of heparin binding and 24 proteins with no evidence and no expectation to bind heparin) using the SPASM algorithm. The results obtained show that, heparin-binding proteins are certainly enriched in the motif (Figure 3).

### CPC clip motif in other heparin-binding proteins

A major limitation in identifying potential GAG binding domains lies in the low degree of sequence conservation. Additionally, some proteins, e.g., chemokines, are known to oligomerize diversifying recognition sites [20], [21]. Limitations are also observed on the GAG side, where despite some particular GAG-binding preference being often found, other GAGs are also recognized, altogether defining a scenario of low-conserved patterns and limited specificity [22]. Analysis of amino acid conservation for the proteins in our study shows that only average-to-low conservation is found for residues involved in heparin binding (Figure S2), stressing the limitations on defining sequence-based patterns for GAG binding sites. As an exception, CPC motifs were found fairly well conserved in enzymes, often close to the catalytic site, suggesting a putative active role of CPC residues in GAG fastening.

#### 1. Patterns for heparin binding in the chemokine family

Chemokines are small proteins that control leukocyte migration during routine immunosurveillance, inflammation, wound healing and angiogenesis [23], [24]. All the chemokines tested have been reported to bind heparin and thus can be referenced as GAG-binding proteins. They can be divided into four classes, all of them sharing a characteristic fold according to its disulfide bridge pattern (CCL, CXCL, CX3CL and CL; according to the International Union of Immunological Societies/World Health Organization Subcommittee on Chemokine Nomenclature [25]). Furthermore, many chemokines form oligomers, a behavior found relevant for their *in-vivo* function [20], [21].

Sequence alignments for both CXCL and CCL chemokines show low similarity, with an average value between 20 to 25% (Figures S3 and S4). In CCL chemokines, a Cardin-Weintraub motif XBBXBX is fairly well conserved. Nonetheless, X-ray diffraction analysis on CCL5 (PDB code 1U4L) showed that additional residues are also fundamental for heparin binding (Figure S5) [26]. Also, in CCL2, four additional cationic residues (Arg18, Lys19, Arg24 and Lys58), not located in the Cardin-Weintraub motif have been found necessary [27].

In contrast, there is no conserved Cardin-Weintraub motif in the CXCL chemokine family, only a XBBXBX motif in CXCL12. Even in this case, other residues located farther away from the motif have been shown to take part in heparin recognition (PDB code 2NWG; Figure S3) [28].

The CPC clip motif is well defined in both CCL5 and CXCL12 crystal structures and appears to be preserved throughout the whole CCL and CXCL chemokine families (Figures S5 and S6). Modeling simulations were used for all proteins with no structural data in the PDB. Residues not included in the Cardin-Weintraub motif appear well conserved (particularly in the CXCL family), hence supporting the hypothesis that structural patterns are actually contributing. Cα distances are also preserved in the case of CXCL chemokine family, with measured values (6.9±2.3 Å, 12.0±1.6 Å, 14.8±2.3 Å) within the set reference intervals. For CC chemokines, distances appear to be well preserved although mean values (10.9±1.9 Å, 17.2±2.5 Å, 23.9±3.1 Å) differ considerably from the reference ones, a deviation that may come from inherent lack of precision of protein models. Indeed, for CXC chemokines CPC residues are mainly located in regions with defined secondary structure whereas in the CC series, CPC residues are found in highly flexible loops, difficult to evaluate by *in-silico* protein modeling.

Lymphotactin (Ltn, XCL1/XCL2), the representative member of the C chemokine family, involved in the recruitment of T and NK cells [29], has a single disulfide bond and is conformationally heterogeneous, switching between a conserved chemokine fold, named Ltn10 [30], and an unrelated dimeric structure, Ltn40 [31]. This reversible interconversion alters the heparin-binding site allowing Ltn40 to bind tighter to heparin than Ltn10 [32].

**Figure 4:**
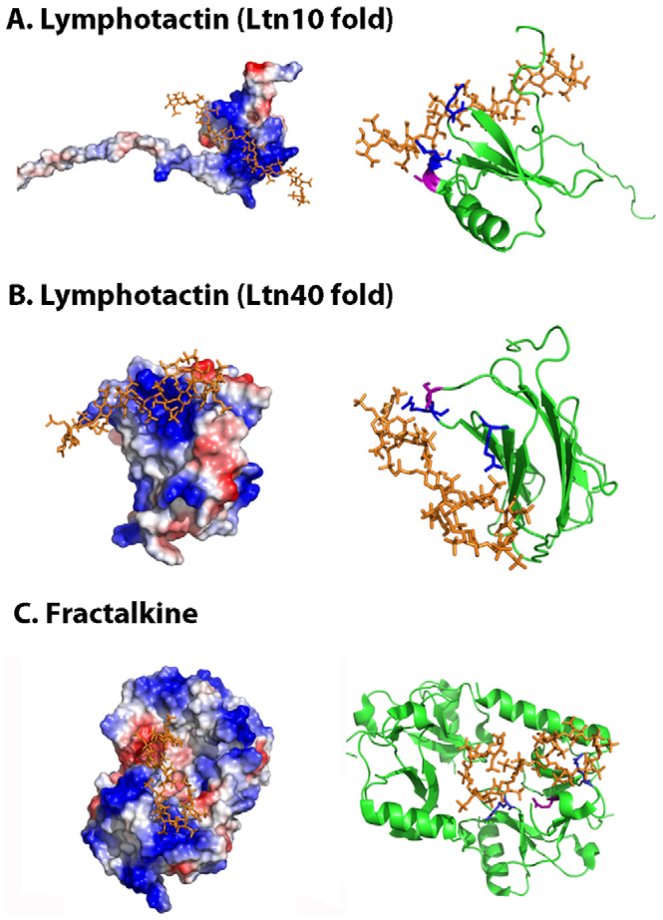
Molecular docking simulation of lymphotactin and fractalkine heparin-binding sites. The figure displays the protein electrostatic potential (left) and the protein cartoon highlighting in red the CPC clip motif (right) of lymphotactin Ltn10 (A) and Ltn40 (B) and CDF fractalkine domain (C). CPC residues are colored in blue (cationic) and magenta (polar). Heparin dodecasaccharide ligand used in docking simulations is colored in orange. PDB codes: 1J8I (Ltn10), 2JP1 (Ltn40), 1B2T (fractalkine) and 1HPN (heparin ligand).

Since a structure for Ltn complexed to heparin or heparin derivatives is unavailable, we have conducted molecular docking simulations (using 1HPN heparin dodesaccharide as a ligand) to find putative heparin binding sites. Our results (Figure 4A,B) suggest that both, the Ltn10 (PDB code 1J8I) and Ltn40 (PDB code 2JP1) can tightly bind heparin (Table 1) through a cationic surface, as described for most chemokines. In this model, Arg and Lys residues provide the main interactions (Arg23, Lys25, Lys42, Arg 43, Lys46, Lys66) and one polar residue (Ser22) is close enough to make hydrogen-bonding contact with the ligand. These results are supported both by NMR and heparin-sepharose chromatography. Particularly, Arg23 and Arg43 are the ones undergoing stronger NMR chemical shifts upon heparin binding [33] and can be viewed as defining a CPC clip motif together with the polar residue Ser22 in both folds (Figure 4A,B), with Cα distances (3.8 Å, 11.7 Å and 8.4 Å for Ltn10 and 3.8 Å, 11.7 Å and 12.0 Å for Ltn40) consistent with reference values.

**Table 1:** Average binding energies for the docked heparin-protein complexes^a^.

PDB code	CPC motif	Average free energy of binding (kcal/mol)^b^
1F2L	Gln31, Arg37, Arg47	−9.25/−5.95
1J8I	Ser22, Arg23, Arg43	−18.41/−8.29
1MWP	Thr59, Arg100, Arg102	−4.22/−4.91
1TKN	Asn475, Arg468, Lys496	−9.95/−9.21
2JP1	Ser22, Arg23, Arg43	−20.04/−7.54

a All docking simulations have been carried out using Autodock with a heparin dodesaccharide or disaccharide allowing ten or two degrees of freedom respectively (Figure S1). See *Materials and Methods* section for further information.

b First value refers to dodesaccharide docking whereas second value refers to the disaccharide.

Finally, the single member of the CX3C chemokine class, named chemical domain of fractalkine (CDF or CX3CL1) and involved in the capture and activation of leukocytes [34], binds to heparin with similar strength as other chemokines and displays a similar electrostatic surface [35]. There is no structure of CDF complexed to heparin mimetics, thus we have again resorted to docking simulations. As shown in Figure 4C, the binding surface of CDF is highly cationic and comprises several lysine residues, (Arg37, Arg47, Lys 59). Again, a CPC clip motif can be described in this surface (Table 1), comprising residues Gln31, Arg37 and Arg47, with measured distances 7.7 Å, 11.6 Å and 16.8 Å.

In summary, the CPC clip motif has been found to correctly describe the heparin-binding sites of chemokines, providing additional clues on the characterization of discontinuous motives.

#### 2. New insights into the heparin-binding site of human amyloid β protein

The extracellular accumulation of amyloid β proteins in neuritic plaques is one of the hallmarks of Alzheimer's disease [36]. Amyloid Aβ protein can bind to many macromolecules, including heparin. Although Aβ can self-aggregate to form amyloid fibrils *in vitro*, its binding to heparin enhances amyloid aggregation and fibril formation. It has been shown that the sulfate moiety is necessary for the growing of amyloid aggregates, as no fibrils are observed in the presence of hyaluronic acid (HA), a non-sulfated GAG [37]. Low-molecular-weight heparins (LMWHs) can reverse the process of amyloidosis, by inhibiting fibril formation and blocking the formation of β-plated structures, underlining a possible therapeutic approach [38].

**Figure 5:**
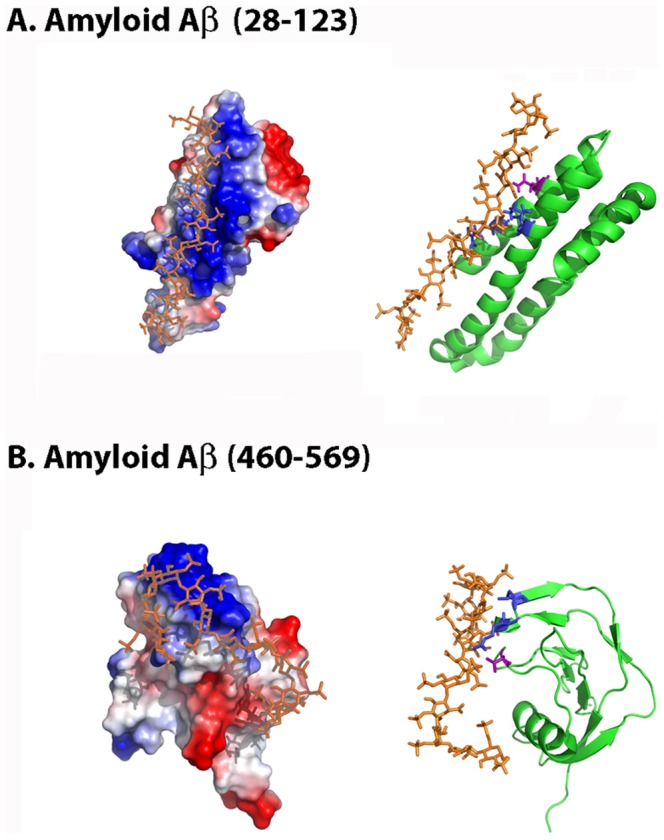
Molecular docking simulation of Aβ28–123 and Aβ460–569 heparin-binding sites. The figure displays the protein electrostatic potential (left) and the protein cartoon highlighting in red the CPC clip motif (right) of (A) Aβ28–123 and (B) Aβ460–569. CPC residues are colored in blue (cationic) and magenta (polar). Heparin dodecasaccharide ligand used in docking simulations is colored in orange. PDB codes: 1MWR (Aβ28–123), 1TKN (Aβ460–569) and 1HPN (heparin ligand).

We have assessed the binding surface of Aβ to heparin by molecular docking simulation on two crystallized fragments of Aβ (Aβ28–123, PDB code 1MWR and Aβ460–569, PDB code 1TKN), both found to bind heparin [39], [40]. As in the case of chemokines and other heparin-binding proteins, both regions are highly cationic (Figure 5).

In Aβ28–123, the main interacting residues correlate with a Cardin-Weintraub motif XBBXBX (namely ^98^CKRGRK^103^) found critical for heparin binding [39]. However, other polar residues that contribute to heparin binding (Asn46, His44, Ser54, Ser57 and Thr59) are also found, and a CPC clip motif defined by residues Thr59, Arg100 and Arg102, with distance values of 5.7 Å, 8.0 Å and 6.0 Å can be proposed for this region, the last value however found out from the reference values.

Our docking studies also suggest that the Aβ460–569 region, reported as crucial for heparin binding [40], contains a CPC clip motif defined by residues Arg468, Asn475 and Lys496 (distances 8.9 Å, 10.3 Å and 10.2 Å). Other residues in this region found to interact are Thr478, His489, Arg 495, Arg499, Lys503 and Lys510. These residues do not define any Cardin-Weintraub motif, despite the importance of the region for heparin binding. In particular, our docking results suggest that the binding site located in the Aβ460–569 region would bind tightly heparin whereas the site located in Aβ28–123 could be a complementary binding site (Table 1).

### Searching for other proteins containing the CPC clip motif

By means of the SPASM algorithm, we have analyzed the PDB database to find the described CPC motif in proteins reported to bind heparin but for which no structural information on their binding domain is available. SPASM uses a fast search process based on differences between atomic positions. We have analyzed the five canonic CPC motifs identified (i.e., C-Asn-C, C-Gln-C, C-Ser-C, C-Thr-C and C-Tyr-C) to find other proteins potentially able to bind heparin. However, detection of small structural motifs is complex and sometimes lacks specificity due to the size of the database. To further refine our search, SAVANT has been used to perform an all-atom least squares superpositioning of the CPC pattern and the SPASM hits. We used CD-HIT to avoid including similar proteins and PANTHER to analyze and classify the function of proteins identified by SPASM. From these proteins, the main portion is related to binding proteins and enzymes (>75%). Around 20% are related to primary metabolism, from which a 40% is dedicated to metabolites containing sugars derivatives. Regarding function, many of them have characteristic roles found in heparin-binding proteins like cell communication, adhesion and/or proliferation (Figure S7).

While the above method could be useful to detect new proteins with no previously reported heparin binding, it tends to unveil a large number of candidates that require subsequent filtering, and therefore is only suitable as a complementary searching tool. In conclusion, we have found a structural motif conserved in heparin-binding proteins that provide a cationic surface environment to fix heparin. This motif would act as a staple for polymeric GAG substrates and provides useful clues on why GAG binding proteins display considerable sequence diversity.

## Supporting Information

Figure S1 **Representation of heparin dodesaccharide (A, PDB code 1HPN) and disaccharide (B, PDB code 1U4M) molecules used in docking simulations.** Allowed torsions in the simulation are colored in red whereas fixed bonds are colored in green.
(JPG)

Figure S2 **Representation of the amino acid conservation for the 20 reference protein-heparin complexes.** Ligands are colored in orange and amino acid residues are colored by conservation as depicted in the scale provided at the bottom of the image; residues were colored in yellow when now enough information was available. Images were computed using Consurf and generated with *Pymol.*
(JPG)

Figure S3 **Sequence alignment of human CXCL chemokines.** Conserved residues are colored in red; the putative XBBXBX motif is highlighted in orange. Green arrows indicate the two CPC clip motives found in CXCL chemokines.
(JPG)

Figure S4 **Sequence alignment of human CCL chemokines.** Conserved residues are colored in red; the putative XBBXBX motif is highlighted in orange. Green arrows indicate the CPC clip motif found in CCL chemokines.
(JPG)

Figure S5 **Molecular representation of the CPC clip motif for the CXCL chemokine complexes.** For each complex, the ligand is colored in blue, the amino acids belonging to the reference CPC clip motif (CXCL12; PDB code 2NWG) are colored in red and the CPC residues corresponding to the modeled chemokine are colored in green. Residues not matching with the CPC description are colored in olive green. Images were generated with Pymol.
(JPG)

Figure S6 **Molecular representation of the CPC clip motif for the CCL chemokine complexes.** For each complex, the ligand is colored in blue, the amino acids belonging to the reference CPC clip motif (CCL5; PDB code 1U4L) are colored in red and the CPC residues corresponding to the modeled chemokine are colored in green. Residues not matching with the CPC description are colored in olive green. Images were generated with Pymol.
(JPG)

Figure S7 **Function distribution of the genes that encode for the putative heparin-binding regions detected by SPASM.** The list of proteins obtained by SPASM (PDB codes) was translated to a SwissProt identifier list using PICR (http://www.ebi.ac.uk/Tools/picr/). The database generated was inspected using PANTHER (http://www.pantherdb.org/) and the genes identified were classified by GO annotation.
(JPG)

Table S1 **List of proteins included in the reference data set.**
(DOCX)
