# Supplementary figures and images for: The “CPC Clip Motif”: A Conserved Structural Signature for Heparin-Binding Proteins
Source: PLoS One. 2012 Aug 6;7(8):e42692. doi: 10.1371/journal.pone.0042692 (PMC3412806; doi:10.1371/journal.pone.0042692)

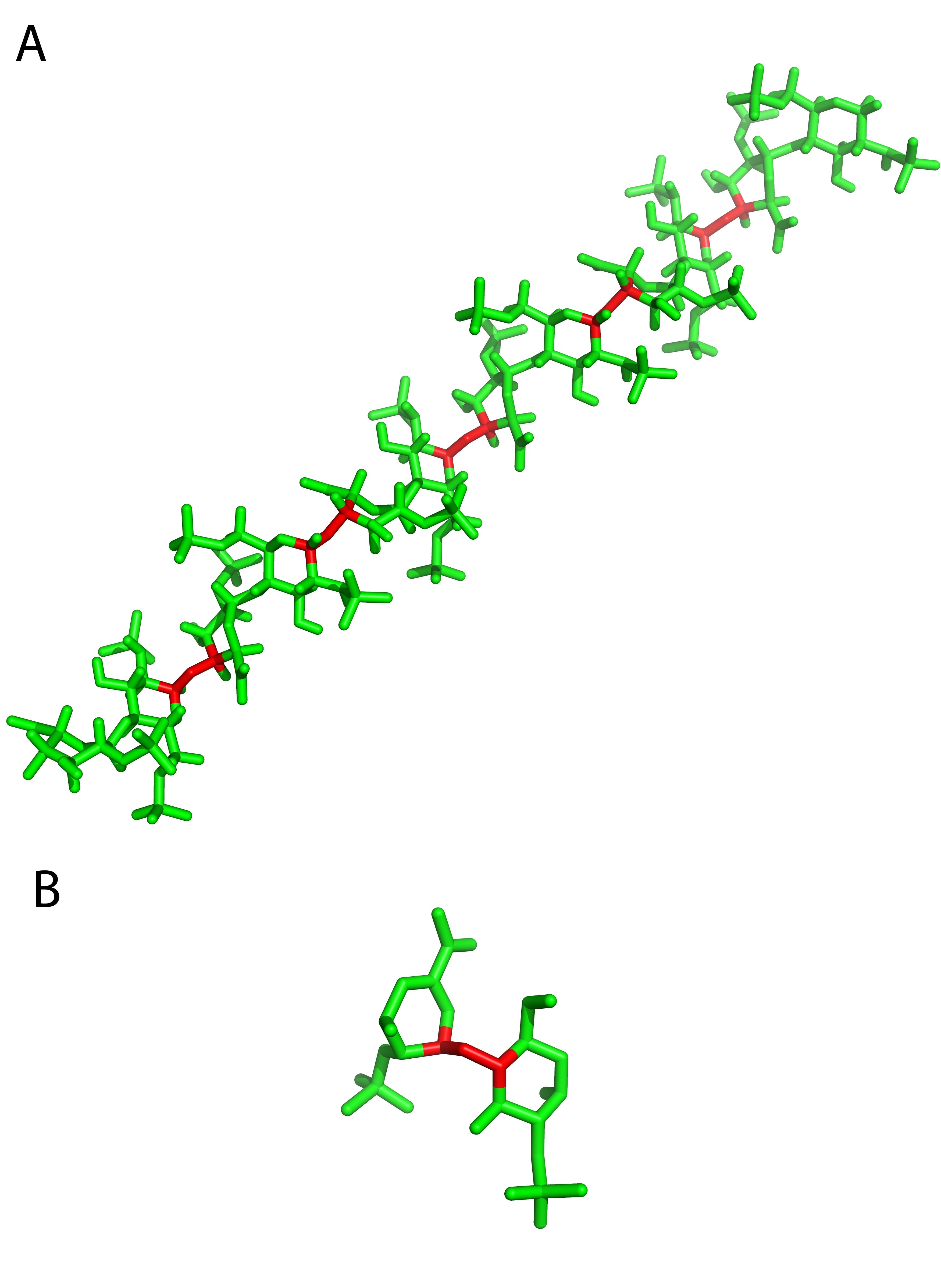

Supplement: Figure S1 — Representation of heparin dodesaccharide (A, PDB code 1HPN) and disaccharide (B, PDB code 1U4M) molecules used in docking simulations. Allowed torsions in the simulation are colored in red whereas fixed bonds are colored in green. (JPG) [file pone.0042692.s001.jpg]

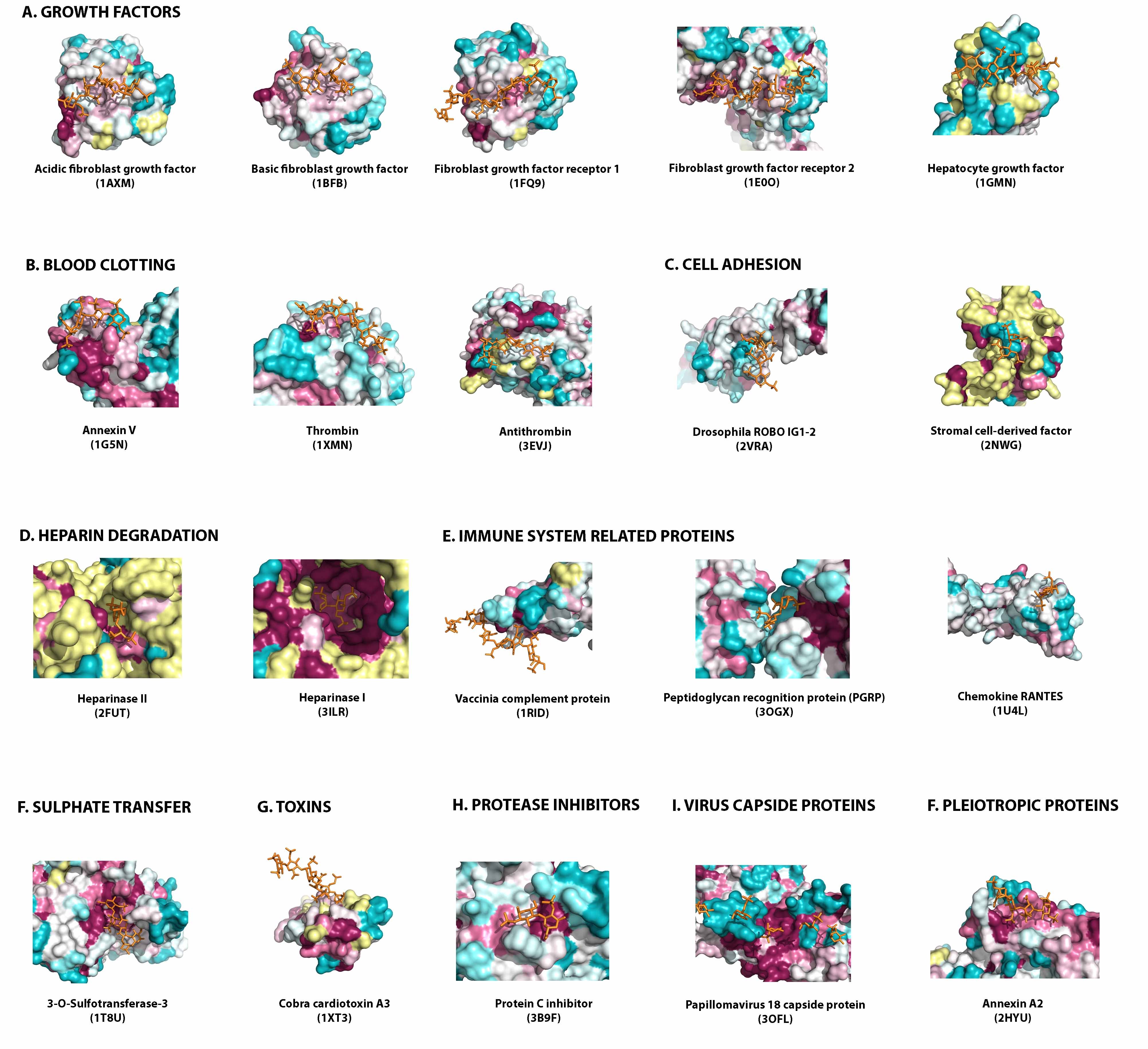

Supplement: Figure S2 — Representation of the amino acid conservation for the 20 reference protein-heparin complexes. Ligands are colored in orange and amino acid residues are colored by conservation as depicted in the scale provided at the bottom of the image; residues were colored in yellow when now enough information was available. Images were computed using Consurf and generated with Pymol. (JPG) [file pone.0042692.s002.jpg]

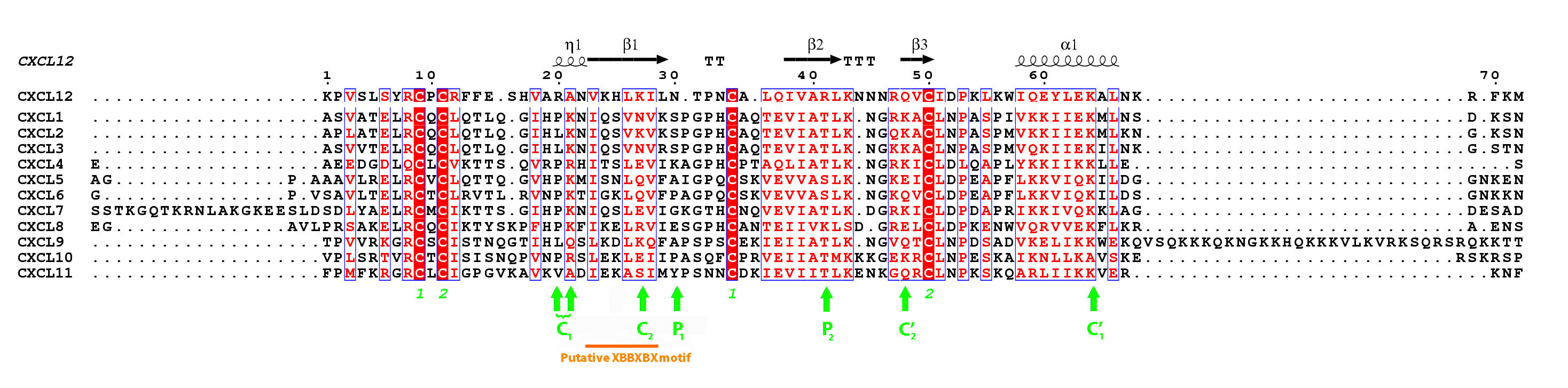

Supplement: Figure S3 — Sequence alignment of human CXCL chemokines. Conserved residues are colored in red; the putative XBBXBX motif is highlighted in orange. Green arrows indicate the two CPC clip motives found in CXCL chemokines. (JPG) [file pone.0042692.s003.jpg]

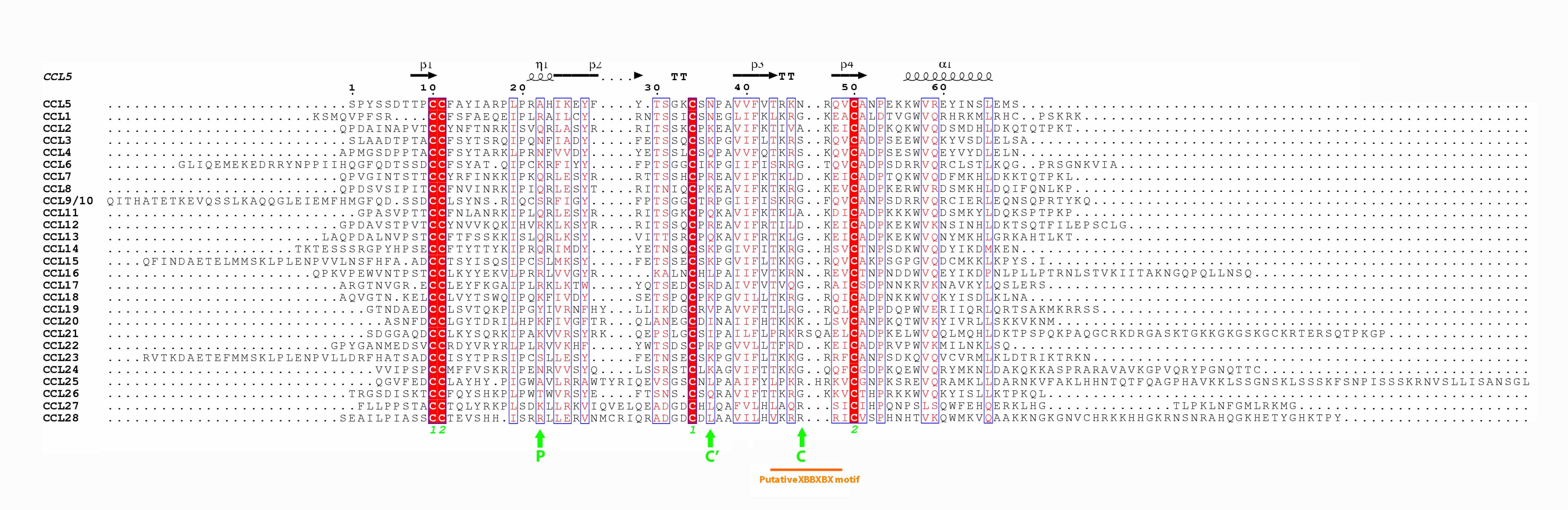

Supplement: Figure S4 — Sequence alignment of human CCL chemokines. Conserved residues are colored in red; the putative XBBXBX motif is highlighted in orange. Green arrows indicate the CPC clip motif found in CCL chemokines. (JPG) [file pone.0042692.s004.jpg]

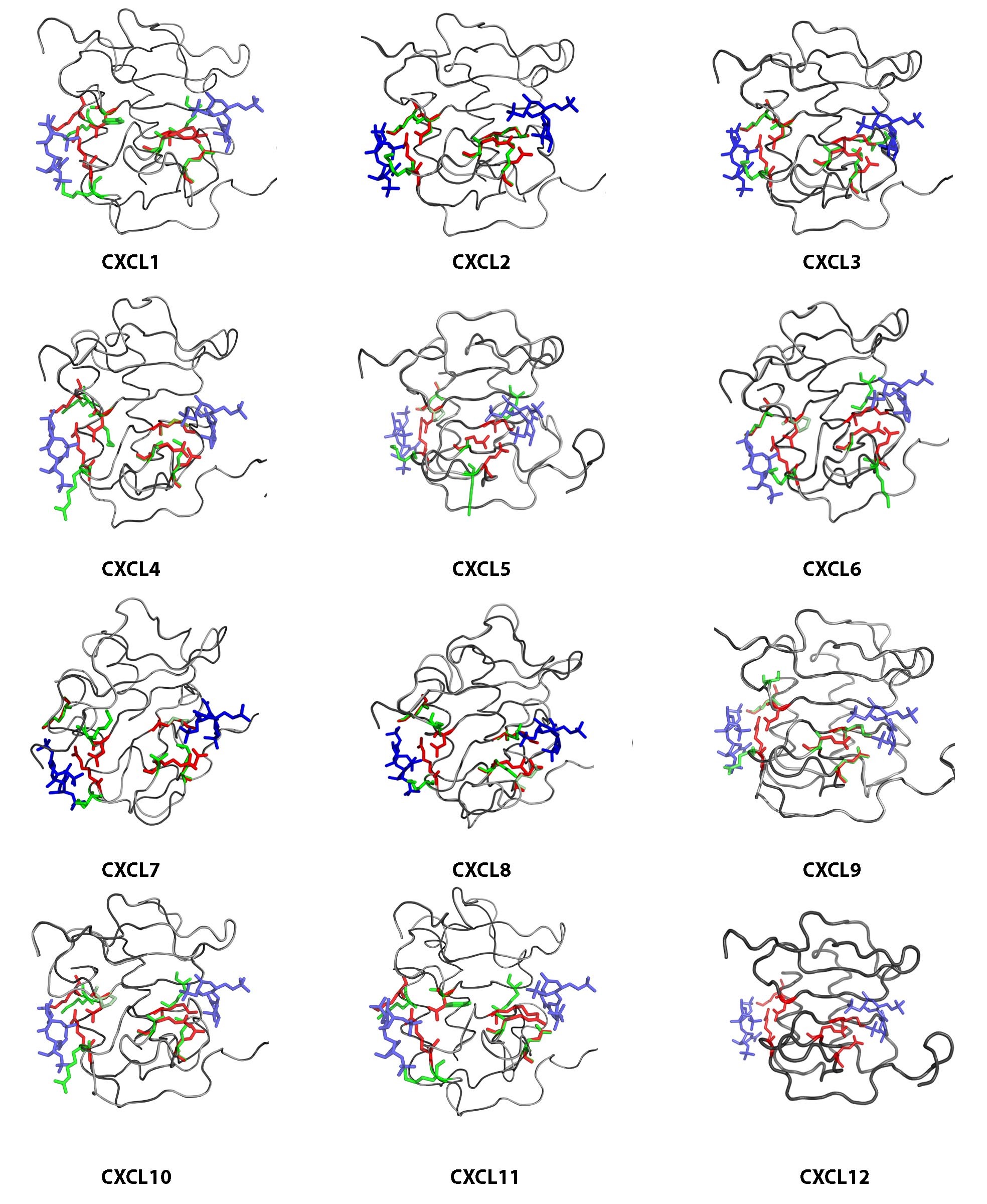

Supplement: Figure S5 — Molecular representation of the CPC clip motif for the CXCL chemokine complexes. For each complex, the ligand is colored in blue, the amino acids belonging to the reference CPC clip motif (CXCL12; PDB code 2NWG) are colored in red and the CPC residues corresponding to the modeled chemokine are colored in green. Residues not matching with the CPC description are colored in olive green. Images were generated with Pymol. (JPG) [file pone.0042692.s005.jpg]

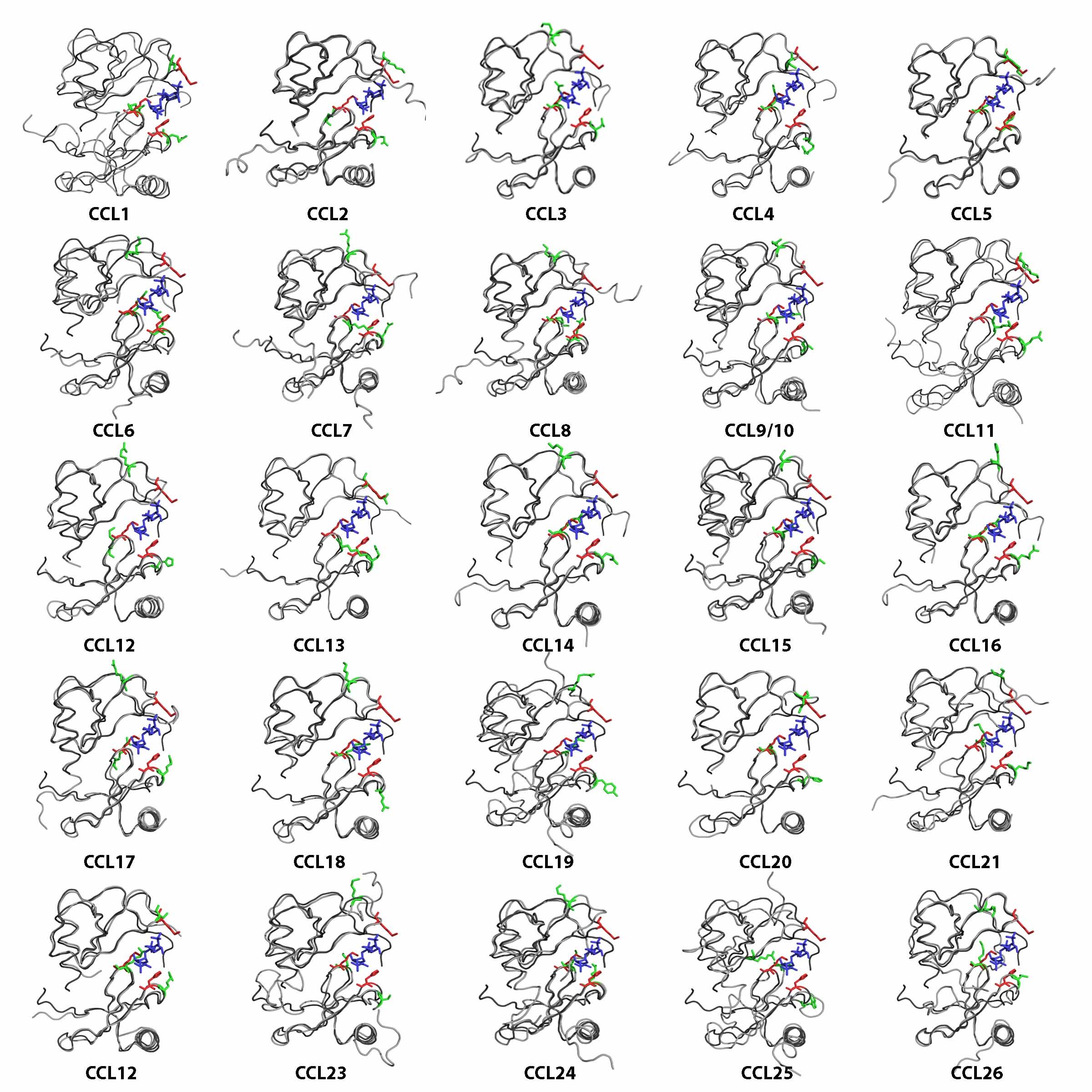

Supplement: Figure S6 — Molecular representation of the CPC clip motif for the CCL chemokine complexes. For each complex, the ligand is colored in blue, the amino acids belonging to the reference CPC clip motif (CCL5; PDB code 1U4L) are colored in red and the CPC residues corresponding to the modeled chemokine are colored in green. Residues not matching with the CPC description are colored in olive green. Images were generated with Pymol. (JPG) [file pone.0042692.s006.jpg]

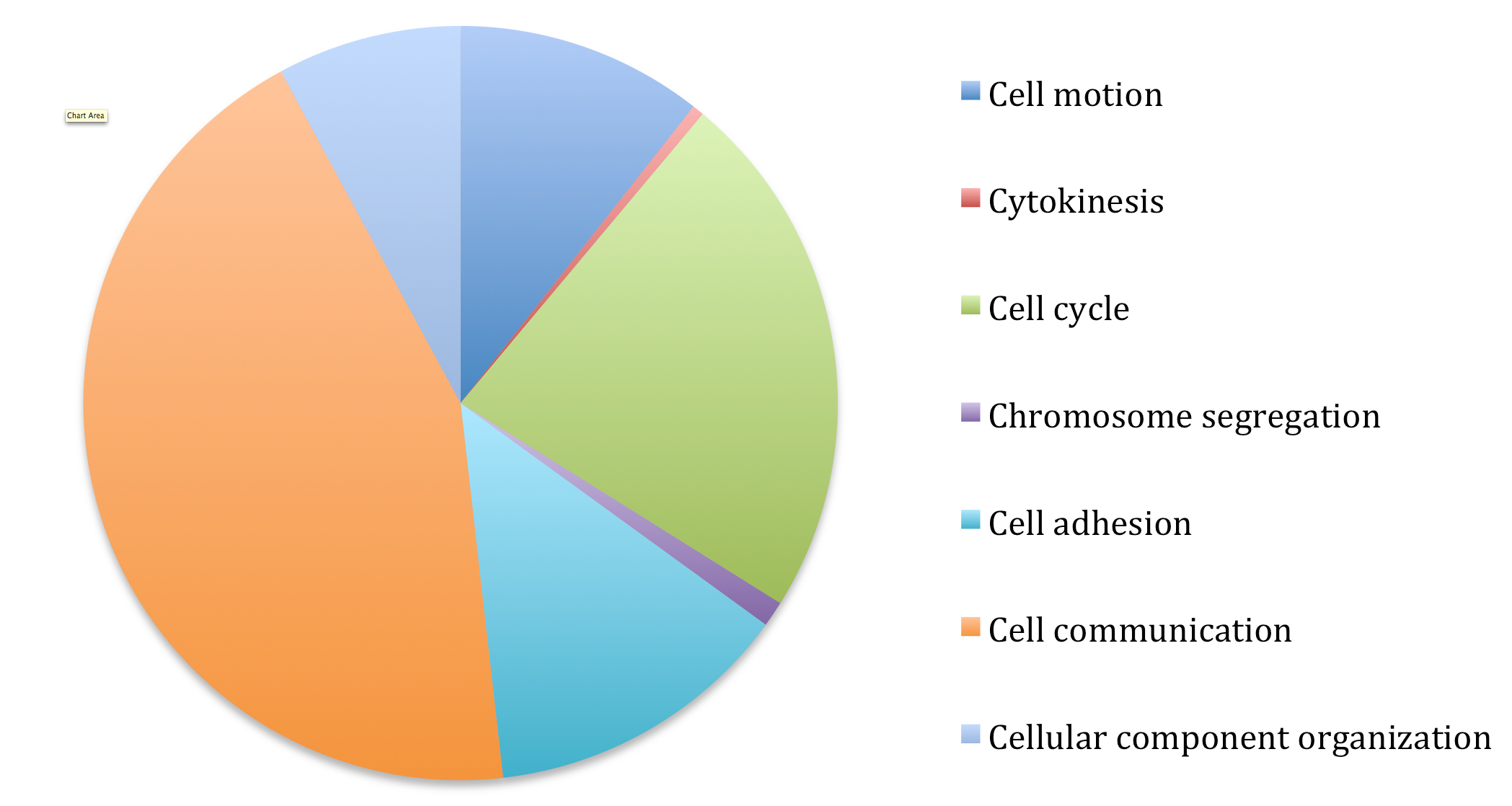

Supplement: Figure S7 — Function distribution of the genes that encode for the putative heparin-binding regions detected by SPASM. The list of proteins obtained by SPASM (PDB codes) was translated to a SwissProt identifier list using PICR (http://www.ebi.ac.uk/Tools/picr/). The database generated was inspected using PANTHER (http://www.pantherdb.org/) and the genes identified were classified by GO annotation. (JPG) [file pone.0042692.s007.jpg]
