# Supplementary material for: The “CPC Clip Motif”: A Conserved Structural Signature for Heparin-Binding Proteins
Source: PLoS One. 2012 Aug 6;7(8):e42692. doi: 10.1371/journal.pone.0042692 (PMC3412806; doi:10.1371/journal.pone.0042692)
Supplement: Table S1 — List of proteins included in the reference data set. (DOCX) [file pone.0042692.s008.docx]

**TABLE S1.** List of proteins included in the reference data set.

| **Protein name** | **PDB code** | **Size^a^** | **Heparin affinity ^b^** | **Reference** |
| --- | --- | --- | --- | --- |
| Acidic fibroblast growth factor | 1AXM | 135 | 1.7 nM | [[1](#_ENREF_1)] |
| Basic fibroblast growth factor | 1BFB | 147 | 5.0 nM | [[1](#_ENREF_1)] |
| Fibroblast growth factor receptor 1 | 1FQ9 | 225 | 400 nM^c^ | [[2](#_ENREF_2)] |
| Fibroblast growth factor receptor 2 | 1E0O | 219 | 400 nM^c^ | [[2](#_ENREF_2)] |
| Hepatocyte growth factor | 1GMN | 183 | 12nM | [[3](#_ENREF_3)] |
| Annexin V | 1G5N | 318 | 21 mM | [[4](#_ENREF_4)] |
| Thrombin | 1XMN | 259 | 1000 nM | [[5](#_ENREF_5)] |
| Antithrombin | 3EVJ | 432 | 1.3 nM | [[6](#_ENREF_6)] |
| Drosophila ROBO IG1-2 | 2VRA | 208 | n.d. | - |
| Stromal cell-derived factor CXCL12 | 2NWG | 68 | n.d. | - |
| Heparinase 1 | 3ILR | 370 | 60 nM | [[7](#_ENREF_7)] |
| Heparinase 2 | 2FUT | 748 | n.d. | - |
| Vaccinia complement protein | 1RID | 244 | 125 nM | [[8](#_ENREF_8)] |
| Peptidoglycan recognition protein | 3OGX | 171 | n.d. | - |
| Chemokine RANTES CCL5 | 1U4L | 68 | 32.1 nM | [[9](#_ENREF_9)] |
| 3-O-sulfotransferase-3 | 1T8U | 272 | n.d. | - |
| Cobra cardiotoxin A3 | 1XT3 | 60 | 2250 nM | [[10](#_ENREF_10)] |
| Protein C inhibitor | 3B9F | 395 | n.d. | - |
| Papillomavirus 18 capsid protein | 3OFL | 427 | 75.9 nM | [[11](#_ENREF_11)] |
| Annexin A2 | 2HYU | 308 | 1.7 nM | [[12](#_ENREF_12)] |

^a^ in number of residues

^b^ n.d., no description available

^c^ values based on fibroblast growth factor receptor 4

**References:**

1. Cochran S, Li CP, Ferro V (2009) A surface plasmon resonance-based solution affinity assay for heparan sulfate-binding proteins. Glycoconj J 26: 577-587.

2. Saxena K, Schieborr U, Anderka O, Duchardt-Ferner E, Elshorst B, et al. (2010) Influence of heparin mimetics on assembly of the FGF.FGFR4 signaling complex. J Biol Chem 285: 26628-26640.

3. Ashikari-Hada S, Habuchi H, Kariya Y, Itoh N, Reddi AH, et al. (2004) Characterization of growth factor-binding structures in heparin/heparan sulfate using an octasaccharide library. J Biol Chem 279: 12346-12354.

4. Capila I, VanderNoot VA, Mealy TR, Seaton BA, Linhardt RJ (1999) Interaction of heparin with annexin V. FEBS Lett 446: 327-330.

5. Qureshi SH, Yang L, Manithody C, Iakhiaev AV, Rezaie AR (2009) Mutagenesis studies toward understanding allostery in thrombin. Biochemistry 48: 8261-8270.

6. Richard B, Swanson R, Olson ST (2009) The signature 3-O-sulfo group of the anticoagulant heparin sequence is critical for heparin binding to antithrombin but is not required for allosteric activation. J Biol Chem 284: 27054-27064.

7. Sasisekharan R, Venkataraman G, Godavarti R, Ernst S, Cooney CL, et al. (1996) Heparinase I from Flavobacterium heparinum. Mapping and characterization of the heparin binding domain. J Biol Chem 271: 3124-3131.

8. Shih PC, Yang MS, Lin SC, Ho Y, Hsiao JC, et al. (2009) A turn-like structure "KKPE" segment mediates the specific binding of viral protein A27 to heparin and heparan sulfate on cell surfaces. J Biol Chem 284: 36535-36546.

9. Martin L, Blanpain C, Garnier P, Wittamer V, Parmentier M, et al. (2001) Structural and functional analysis of the RANTES-glycosaminoglycans interactions. Biochemistry 40: 6303-6318.

10. Patel HV, Vyas AA, Vyas KA, Liu YS, Chiang CM, et al. (1997) Heparin and heparan sulfate bind to snake cardiotoxin. Sulfated oligosaccharides as a potential target for cardiotoxin action. J Biol Chem 272: 1484-1492.

11. Joyce JG, Tung JS, Przysiecki CT, Cook JC, Lehman ED, et al. (1999) The L1 major capsid protein of human papillomavirus type 11 recombinant virus-like particles interacts with heparin and cell-surface glycosaminoglycans on human keratinocytes. J Biol Chem 274: 5810-5822.

12. Shao C, Zhang F, Kemp MM, Linhardt RJ, Waisman DM, et al. (2006) Crystallographic analysis of calcium-dependent heparin binding to annexin A2. J Biol Chem 281: 31689-31695.
